# Genomic insights into the pathogenesis of Epstein–Barr virus-associated diffuse large B-cell lymphoma by whole-genome and targeted amplicon sequencing

**DOI:** 10.1038/s41408-021-00493-5

**Published:** 2021-05-26

**Authors:** Niklas Gebauer, Axel Künstner, Julius Ketzer, Hanno M. Witte, Tobias Rausch, Vladimir Benes, Jürgen Zimmermann, Judith Gebauer, Hartmut Merz, Veronica Bernard, Lana Harder, Katharina Ratjen, Stefan Gesk, Wolfgang Peter, Yannik Busch, Peter Trojok, Nikolas von Bubnoff, Harald Biersack, Hauke Busch, Alfred C. Feller

**Affiliations:** 1 Department of Hematology and Oncology, University Hospital of Schleswig-Holstein, Campus Lübeck, 23538 Lübeck, Germany; 2 University Cancer Center Schleswig-Holstein, University Hospital of Schleswig-Holstein, Campus Lübeck, 23538 Lübeck, Germany; 3 Medical Systems Biology Group, University of Lübeck, Ratzeburger Allee 160, 23538 Lübeck, Germany; 4 Institute for Cardiogenetics, University of Lübeck, Ratzeburger Allee 160, 23538 Lübeck, Germany; 5 Department of Pediatrics, University Hospital of Schleswig-Holstein, Campus Lübeck, 23538 Lübeck, Germany; 6 Department of Hematology and Oncology, Federal Armed Hospital Ulm, Oberer Eselsberg 40, 89081 Ulm, Germany; 7 EMBL, European Molecular Biology Laboratory, Genomics Core Facility, Meyerhofstraße 1, 69117 Heidelberg, Germany; 8 Department of Internal Medicine I, University Hospital of Schleswig-Holstein, Campus Lübeck, 23538 Lübeck, Germany; 9 Hämatopathologie Lübeck, Reference Centre for Lymph Node Pathology and Haematopathology, Lübeck, Germany; 10 Institut für Tumorgenetik Nord, Steenbeker Weg 23, 24106 Kiel, Germany; 11 HLA Typing Laboratory of the Stefan-Morsch-Foundation, 557565 Birkenfeld, Germany; 12 Institut für Tranfusionsmedizin, Universitätsklinikum Köln. Kerpenerstr. 62, 50937 Köln, Germany

**Keywords:** Cancer genomics, Cytogenetics

## Abstract

Epstein–Barr virus (EBV)-associated diffuse large B-cell lymphoma not otherwise specified (DLBCL NOS) constitute a distinct clinicopathological entity in the current World Health Organization (WHO) classification. However, its genomic features remain sparsely characterized. Here, we combine whole-genome sequencing (WGS), targeted amplicon sequencing (tNGS), and fluorescence in situ hybridization (FISH) from 47 EBV + DLBCL (NOS) cases to delineate the genomic landscape of this rare disease. Integrated WGS and tNGS analysis clearly distinguished this tumor type from EBV-negative DLBCL due to frequent mutations in *ARID1A* (45%), *KMT2A/KMT2D* (32/30%), *ANKRD11* (32%), or *NOTCH2* (32%). WGS uncovered structural aberrations including 6q deletions (5/8 patients), which were subsequently validated by FISH (14/32 cases). Expanding on previous reports, we identified recurrent alterations in *CCR6* (15%), *DAPK1* (15%), *TNFRSF21* (13%), *CCR7* (11%), and *YY1* (6%). Lastly, functional annotation of the mutational landscape by sequential gene set enrichment and network propagation predicted an effect on the nuclear factor κB (NFκB) pathway (*CSNK2A2*, *CARD10*), IL6/JAK/STAT (*SOCS1/3*, *STAT3*), and WNT signaling (*FRAT1, SFRP5*) alongside aberrations in immunological processes, such as interferon response. This first comprehensive description of EBV + DLBCL (NOS) tumors substantiates the evidence of its pathobiological independence and helps stratify the molecular taxonomy of aggressive lymphomas in the effort for future therapeutic strategies.

## Introduction

Epstein–Barr virus-positive (EBV + ) diffuse large B-cell lymphoma not otherwise specified (DLBCL NOS) was first described in a pivotal study by Oyama et al. in 2003, which reported on 22 senile patients having a predominantly dismal clinical outcome and poor response to established therapeutic regimens. Further retrospective clinicopathological case series led to the introduction of the provisional entity of EBV + DLBCL (NOS) of the elderly in the 2008 version of the World Health Organization (WHO) classification of tumors of hematopoietic and lymphoid tissues. Subsequent studies further refined the perception of EBV + DLBCL (NOS) due to its occasional occurrence in young, immunocompetent individuals with evidence for a tolerogenic immune environment^1–3^. These observations led to an adapted definition of the entity in the 2016 revision of the WHO classification^4,5^.

While EBV-positive patients account for 8–10% of DLBCL cases in the East Asian population, data on the incidence in the western hemisphere is scarce, yet suggests similar to slightly lower numbers. The multifaceted western spectrum of EBV-related lesions, ranging from reactive lymphoid hyperplasia to DLBCL, was, however, found to be phenotypically similar to the spectrum encountered in Asian countries^6–9^.

Although recent observations relativized EBV as an independent risk factor, several studies found EBV + DLBCL (NOS) patients to be significantly enriched for adverse confounding clinical factors, such as stage, impaired performance status, advanced age, and significant canonical and alternative nuclear factor κB (NFκB)-pathway activation^10^. Altogether this resulted in a high number of cases with non-germinal center B-cell-like immunophenotype^2,7,11–14^. These circumstances render the entity a preferable subject for the study of molecularly targeted therapies.

The significant phenotypical overlap between EBV + DLBCL (NOS) and immunodeficiency or transplant-associated lymphoproliferative disorders (LPDs/PTLDs), including similar EBV-latency patterns, was found to be an additional characteristic of the entity^12^.

Intriguingly, NFκB-activation in EBV + DLBCL (NOS) was shown to result from EBV transcriptional reprogramming together with advanced B-cell differentiation^13^. Genomic profiling of MYD88-mediated TLR-signaling, as well as the B-cell receptor signaling pathway, revealed infrequent mutations, unlike the ABC-type DLBCL. Beyond these preliminary observations, the genomic features of EBV + DLBCL (NOS) are hitherto sparsely characterized. An initial study using exome sequencing of EBV + DLBCL (NOS) in a Chinese cohort of eleven patients without matched germline DNA proposed a heterogeneous mutational landscape dominated by a mutational signature associated with failure of DNA double-strand break–repair by homologous recombination^15^.

More recently, a targeted sequencing study of nine patients proposed an elevated frequency of *MYC* and *RHOA* mutations together with other genetic aberrations including mutations in *MEF2B* and *MYD88*^16^. Kataoka et al. addressed the overall impact of an underlying EBV infection on the mutational signature with a predefined 140-gene panel in an extended cohort of different lymphoma subtypes including 27 cases of DLBCL, contrasting their observations with 48 EBV-negative cases^17^. From their study, the authors derived a significant enrichment of mutations in *TET2* and *DNMT3A* in EBV + DLBCL (NOS). Additionally, Sarkozy et al. recently reported whole-exome sequencing data of seven exclusively polymorphic EBV + DLBCL (NOS) with 13 additional cases investigated by panel-based sequencing. Hereby they identified *SOCS1*, *GNA13*, and *CSF2RB* mutations as potential drivers in EBV + DLBCL (NOS) pathogenesis, specifically in its polymorphic subtype, which is morphologically closely related to classical Hodgkin lymphoma^18^.

Although preliminary studies hint at a heterogeneous spectrum of potential genetic drivers in EBV + DLBCL (NOS), a comprehensive characterization of the genomic landscape in a representative cohort of patients is lacking. To this end, we combined whole-genome (*n* = 8) and targeted sequencing approaches (additional 39 patients) with FISH for *MYC*, *BCL2*, and *BCL6* as well as 6q aberrations. Through this integrated analysis, we outline oncogenic drivers, copy number alterations, and pathway perturbations, hereby refining the molecular taxonomy of aggressive B-cell malignancies.

## Materials and methods

### Case selection and clinicopathological characteristics

In a retrospective approach, we reviewed our institutional database to identify EBV + DLBCL (NOS) patients whose primary diagnostic biopsy specimen had been referred to the Reference center for Hematopathology at University Hospital Schleswig Holstein Campus Lübeck and Hämatopathologie Lübeck for centralized histopathological panel evaluation between 2008 and April 2018. From 80 cases meeting diagnostic and clinical criteria for EBV + DLBCL, nine were selected for subsequent whole-genome analysis, based on tumor DNA quality and availability of paired germline DNA. One case was dismissed from analysis due to quality control issues following library preparation. An additional 39 cases with available tumor DNA of sufficient quality were selected for targeted sequencing. Tissue sections for confirmatory FISH diagnostics were available in 32 patients. For further details on clinicopathological workup, see Supplementary Materials and methods as well as Supplementary Table 1. A summary in the format of a flow diagram depicting the selection of cases for the respective analyses alongside a description of drop-outs is provided in Supplementary Fig. 1.

This retrospective study was approved by the ethics committee of the University of Lübeck (reference no. 18-356) and conducted in accordance with the declaration of Helsinki.

### Extraction of nucleic acids

Genomic DNA was extracted from two to four FFPE tissue sections of 5-µm thickness using Maxwell^®^ RSC DNA FFPE kit (Promega, Wiesloch, Germany) or the QiaAmp mini kit 250 (Qiagen, Hilden, Germany), according to the manufacturers’ instructions. DNA samples were subsequently quantified by spectrophotometry utilizing a Nanodrop ND-1000 (Nanodrop^®^).

### Whole-genome sequencing

Whole-genome sequencing (WGS) was performed employing a HiSeq2500 platform (Illumina, San Diego, CA, USA). All samples with successful library preparation (8/9) were taken forward to sequencing. Raw fastq files have been deposited in the European genome-phenome archive (EGA) under the accession number EGAS00001004941.

EBV + DLBCL (NOS) samples were sequenced to a median depth of 30×.

### Variant calling

Raw FASTQ reads were trimmed (adapter and quality values) using fastp^19^ (v0.20.0; minimum length 50 bp, max. unqualified bases 30%, trim tail set to 1), and trimmed reads were mapped to GRCh37/hg19 using bwa mem (v0.7.15)^20^. The resulting *SAM* files were cleaned and sorted and converted into *BAM* format using Picard Tools (v2.18.4). Next, mate-pair information was fixed, duplicates were removed and base quality recalibration was performed using Picard Tools^21^ and dbSNP v138. Single-nucleotide variants (SNVs) and short insertions and deletions (indels) were identified following the best practices for somatic mutations calling provided by GATK^22^. Briefly, GATKs Mutect2^23^ (v4.1.5.0) algorithm was applied to all *BAM* files in tumor-normal matched mode with gnomAD variants as germline resource and the b37 whole-genome panel data as the panel of normal. Afterward, FFPE read orientation artefacts were identified and removed according to GATKs guidelines. Filtered variants were annotated using ANNOVAR^24^ (v2019Oct24). Coverage for reference and alternative alleles for each variant was extracted using vcf-query (VCFtools v0.1.13^25^). The top 20 frequently mutated genes (FLAGS^26^) were removed from further analysis. For further analysis, exonic somatic variants were selected and filtered as follows: minimum coverage of 10, the minimum variant allele frequency of 5%, population allele frequency <0.001 in gnomAD or PopFreqMax database. To identify genes that are more often mutated than expected, MutSigCV (v1.41)^27^ was applied and potential driver genes were identified using *P* < 0.05.

Mutational somatic signatures were estimated genome-wide using variants with at least 10× coverage (total; min cov. For variant 3×). Variants with a variant allele frequency <5% were removed as well. The remaining variants were used to estimate somatic signatures against COSMIC signatures using YAPSA (v1.16.0)^28^. Ploidy and microsatellite stability were assessed applying AMBER, COBALT, and PURPLE on base-recalibrated bam files.

Comparative analysis within the framework of clusters c1–c5 according to Chapuy et al. was performed extracting information about mutated genes and clustering of detected variants.

### Targeted next-generation sequencing

In order to validate the initial delineation of the mutational landscape in EBV + DLBCL (NOS), we employed our in-house custom AmpliSeq panel (Thermo Fisher Scientific, Waltham, MA, USA) for targeted amplicon sequencing (tNGS), encompassing all coding exons of 43 genes (see Supplementary Table 2) in both the discovery cohort as well as an additional expansion cohort of 39 cases. Library preparation was carried out according to the manufacturers’ instructions, and sequencing was performed on the Illumina MiSeq platform (Illumina, San Diego, CA, USA) to a median depth of 2416× (s.d. +/− 1057). Raw fastq files have been deposited in the European genome-phenome archive (EGA) under the accession number EGAS00001004941.

### Panel resequencing data analysis

Resequencing data was processed as described above for whole-genome data, but the remove duplicates step was omitted. Variant calling was done using freebayes (v1.3.2-46-g2c1e395), variants were annotated using ANNOVAR, and coverage for each variant was extracted using vcf-query^29^. Afterward, variants were filtered and only variants with a minimum coverage of 100, minimum variant allele frequency of 5%, population allele frequency <0.001 in gnomAD or PopFreqMax database were kept for further analysis.

### Fluorescence in situ hybridization (FISH) for *MYC*, *BCL2*, *BCL6*, and 6q aberrations

Chromosomal breakpoints were analyzed by means of FISH using commercially available dual-color break-apart probes for 8q24 (*MYC*), 18q21 (*BCL2*), and 3q27 (*BCL6*) (Abbott Vysis, Des Plaines, IL, USA) according to the manufacturer’s instructions as well as a custom-designed (enumeration-)probe targeting 6q deletions found in our exploratory WGS cohort encompassing the genes PRDM1 in the region 6q21 and A20 in the region 6q23 prepared at the Institut für Tumorgenetik Nord.

### Structural variants and copy number variations

Genomic rearrangements were detected using a matched tumor-normal approach following best practices of GRIDSS. The resulting structural variations were filtered using GRIPSS and final variation calls were checked for artefacts^30^. Moreover, in order to detect somatic copy number alterations a fragment-based GC- and mappability correction algorithm was applied, followed by a subsequent analysis of these normalized fragment counts in ~10-kbp windows (window sizes chosen based on the regional coverage)^31^. We hereby screened for deviations from the expected copy number 2 and classified events as gains or losses based on the read depth. For CN2LOH (copy number neutral loss-of-heterozygosity) assessment, we utilized 1000 genomes SNPs that were genotyped across all samples. Results were phased using Eagle2 resulting in haplotype blocks which were then used to average individual SNP B-allele frequencies across the haplotype blocks, thereby essentially “de-noising” of the raw SNP B-allele frequencies in our FFPE samples^32^.

### Network propagation and gene set enrichment analysis

The effect of potential driver genes identified by MutSigCV on neighboring genes was assessed using a network propagation (network diffusion^33^) approach with a regularized Laplacian kernel based on STRINGdb v11^34^ protein–protein interaction network as implemented in the diffuStats R package (v1.10.0)^35^. Mutated genes were set to 1, whereas non-mutated genes were set to 0 to model the behavior of the mutation. Network diffusion was performed using a parametric method with statistical normalization (*z*-scores). Afterward, gene set variation analysis was performed on diffusion scores using the GSVA R package (v1.38.0)^36^ with a Gaussian kernel for continuous values against HALLMARK gene sets (R package msigdf^37^ with minimum/maximum size of the resulting gene sets set to 10/500; MSigDB (v7.1)^38^ and the NFκB-signaling pathway (genes were retrieved from KEGG; entry ID hsa04064). The resulting *z*-scores were used as pre-ranked input for a rank-MANOVA based statistical approach to detect enriched gene sets (mitch R packages)^39,40^.

### Statistical analyses

If not stated differently, all statistical analyses were performed using R (v4.0.3) and tidyverse (v1.3.0)^41^ for data handling. Filtering of genomic regions was performed using the GenomicRanges R (v1.42.0)^42^ package and data was visualized using maftools (v2.6.0)^43^. Progression-free survival and overall survival (PFS, OS) were calculated from the date of diagnosis and censored at the last clinical contact. Survival (PFS and OS) according to potential prognostic factors was estimated by means of the Kaplan–Meier method and univariate log-rank test. Survival analysis was carried out employing the R packages survival (3.2-7) and survminer (v0.4.8).

## Results

### Clinical characteristics of the study group

We collected 47 cases of treatment naive de novo EBV + DLBCL (NOS) with sufficient FFPE material for molecular studies. The median age of the study cohort was 74 years (range 19–90 years). An underlying HIV infection had to be clinically excluded prior to recruitment in all cases and EBV + DLBCL (NOS) tumor cells were shown to express EBER in >50% of large cell infiltrates in accordance with the current WHO definition of the entity. Clinical data retrospectively collected from our institutional database was available in all patients. The majority of patients in our study were male (29/47; 62%) and many presented with advanced-stage disease (23/47 stage III/IV; 49%) and exhibited an adverse prognostic constellation (22/47; 47% R-IPI > 2). Despite their advanced age, most patients received an intensive CHOP-like therapeutic frontline approach (33/47; 70%), resulting in an overall response rate (ORR) of 41/47; 87%.

Baseline characteristics of EBV + DLBCL (NOS) cases included in this study are briefly summarized in Table 1. Most samples of the current study were previously investigated for the impact of treatment variability and clinicopathological baseline characteristics on clinical outcome in EBV + DLBCL (NOS)^10^.

**Table 1:** Baseline clinicopathological characteristics in patients with EBV-positive DLBC.

Characteristics	Whole-genome seq (*n* = 8)	Targeted seq (*n* = 39)	EBV-positive DLBCL (*n* = 47)
Age (yrs.; median + range)	76.5 (68–83)	69.5 (19–90)	74.0 (19–90)
Sex			
Female	2 (25.0%)	16 (41.0%)	18 (38.3%)
Male	6 (75.0%)	23 (59.0%)	29 (61.7%)
*R-*IPI			
0	–	4 (10.3%)	4 (8.5%)
1–2	5 (62.5%)	21 (53.8%)	26 (55.3%)
>2	3 (37.5%)	19 (48.7%)	22 (46.8%)
Stage (Ann Arbor)			
I	1 (12.5%)	6 (15.4%)	7 (14.9%)
II	3 (37.5%)	14 (35.9%)	17 (36.2%)
III	3 (37.5%)	6 (15.4%)	9 (19.1%)
IV	1 (12.5%)	13 (41.0%)	14 (29.8%)
B-Symptoms			
Yes	3 (37.5%)	21 (53.8%)	24 (51.1%)
No	5 (62.5%)	18 (46.2%)	23 (48.9%)
CD30 by immunohistochemistry			
Positive	7 (87.5%)	29 (74.4%)	36 (76.6%)
Negative	1 (12.5%)	10 (25.6%)	11 (23.4%)
Extranodal sites			
0	6 (75.0%)	16 (41.0%)	22 (46.8%)
1–2	2 (25.0%)	23 (59.0%)	25 (53.2%)
*E*COG *PS*			
0–1	5 (62.5%)	15 (38.5%)	20 (42.6%)
≥2	3 (37.5%)	24 (61.5%)	27 (57.4%)
LDH			
Normal	2 (25.0%)	18 (46.2%)	20 (42.6%)
Elevated	6 (75.0%)	21 (53.8%)	27 (57.4%)
CNS involvement at diagnosis			
Yes	–	3 (7.7%)	3 (6.4%)
No	8 (100.0%)	36 (92.3%)	44 (93.6%)
Frontline therapy regimen			
R-CHOP-like	8 (100.0%)	25 (64.1%)	33 (70.2%)
R-based	8 (100.0%)	35 (89.7%)	43 (91.5%)
Others	–	2 (5.1%)	2 (4.3%)
Refusal of treatment	–	1 (2.6%)	1 (2.1%)
Frontline therapy response rates			
CR	7 (87.5%)	23 (59.0%)	30 (63.8%)
PR	1 (12.5%)	10 (25.6%)	11 (23,4%)
SD	–	4 (10.3%)	4 (8.5%)
PD	–	2 (5.1%)	2 (4.3%)

*EBV* Epstein–Barr virus, *Yrs.* years, *CNS* central nervous system, *LDH* lactate dehydrogenase, *ECOG* Eastern cooperative oncology group, *PS* performance status, *CHOP* cyclophosphamide, doxorubicin, vincristine, prednisolone, *R* rituximab, *Others* other regimen (e.g., bendamustine) or palliative cytoreductive treatment.

Baseline characteristics of EBV + DLBCL (NOS) cases included in this study allocated according to affiliation to the discovery or the extension cohort, respectively.

### Genomic landscape of EBV + DLBCL (NOS) patients identified by WGS

**Fig. 1:**
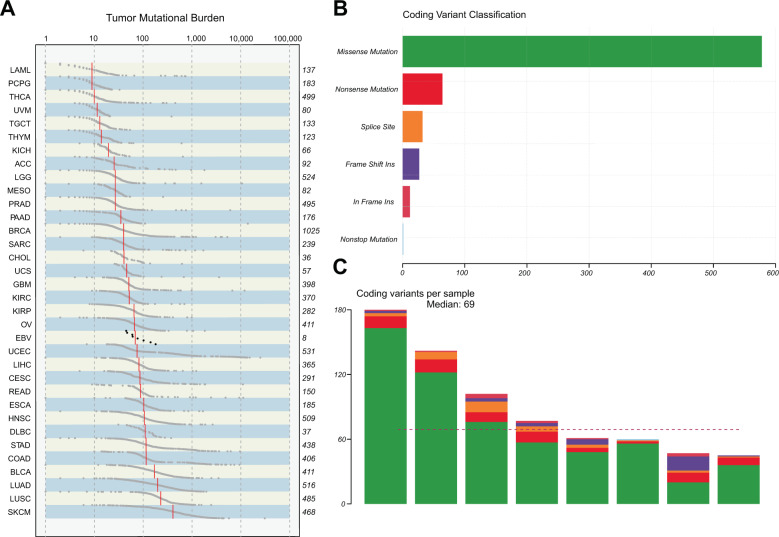
Overview of mutational burden und subtypes. Panel (**A**) shows the mutational burden of EBV + DLBCL (NOS) against TCGA cohorts, the number of coding variants stratified by variant classification is depicted in panel (**B**), and the number of non-silent mutations per sample is shown in (**C**).

**Fig. 2:**
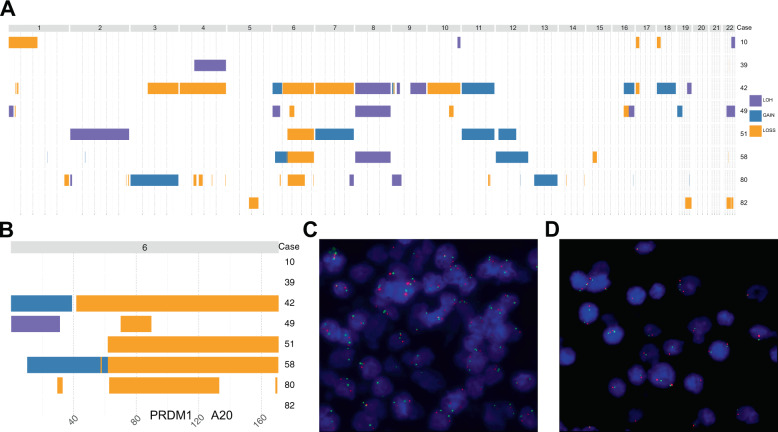
Copy number variants (CNVs) in EBV + DLBCL (NOS) identified through whole-genome sequencing. Panel (**A**) depicts whole genomic CNAs, whereas panel (**B**) illustrates the recurrent 6q losses subsequently validated by FISH (A20 in spectrum orange, PRDM1 in spectrum green) with panel displaying a wild-type constellation (**C**) and panel showing a case with a deletion of the A20 and PRDM1 gene locus in 6q (**D**).

**Fig. 3:**
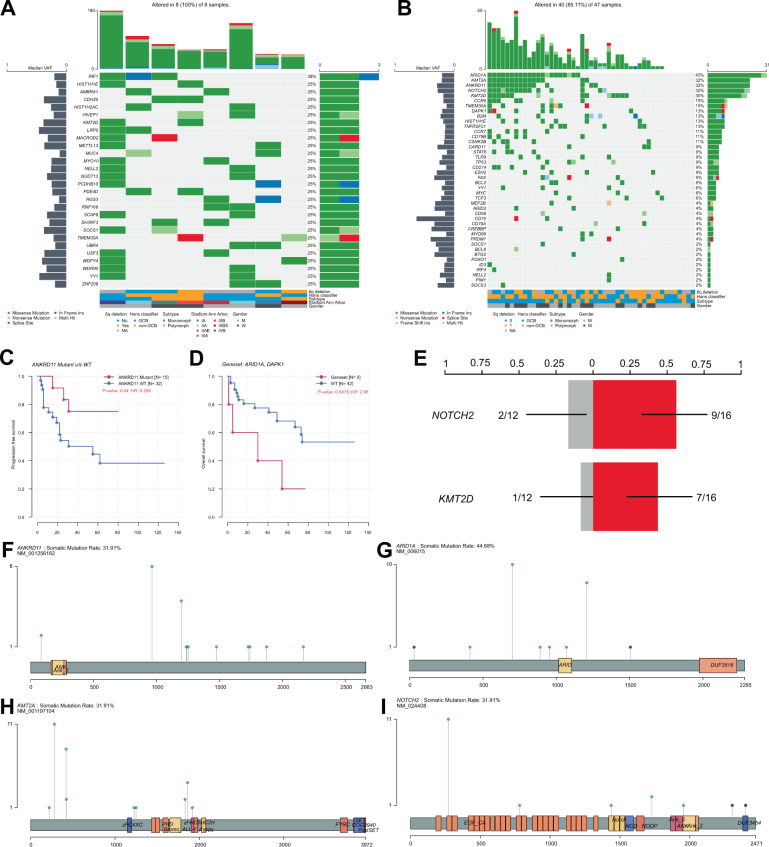
Mutational signature of EBV+ DLBCL (NOS). Panel (**A**) depicts an oncoplot of all genes harboring mutations in at least two samples of our discovery WGS cohort. Panel (**B**) illustrates the mutational landscape in our discovery and extension cohort of EBV + DLBCL (NOS) by tNGS. Mutations in *ANKRD11* were identified as indicators of superior relapse-free but not overall survival (**C**). Moreover, distinct molecular subtypes exhibiting combined alterations of *ARID1A* and *DAPK1* are shown to exhibit inferior overall survival (**D**). The corresponding endpoint analysis for (**C**) and (**D**) (overall survival for ANKRD11 mutation status and progression-free survival for our combined mutation set of ARID1A and DAPK1) is provided in Supplementary Fig. 7. For information on outcome according to these two mutational markers in the subset of R-CHOP treated patients, please see Supplementary Fig. 8. Further, we found mutations in both *KMT2D* as well as *NOTCH2* to be significantly enriched in 6q wild-type patients (**E**). Lollipop plots illustrating the localized distribution of mutational patterns affecting *ARID1A*, *KMT2A*, *ANKRD11*, and *NOTCH2* in our combined cohorts; green dots refer to missense mutations; gray to truncating mutations (**F**–**I**).

To delineate the entire spectrum of genetic alterations in this rare subtype of aggressive B-cell lymphoma, we performed WGS of paired tumor and normal samples from eight EBV + DLBCL (NOS) patients. Clinicopathological baseline characteristics of these specific patients are summarized in Table 1. We identified single-nucleotide variants and indels in individual samples after applying a filtering algorithm to correct for FFPE-derived artefacts and spurious mutations (“Methods”). All EBV + DLBCL (NOS) cases harbored mutations in genes implicated in oncogenesis according to our bioinformatic annotations^44^. We hereby detected on average 89 exonic somatic mutations (median: 69; resulting in a low tumor mutational burden of 2.318 mutations/Mb/sample on average), including 675 SNVs and 39 insertions and deletions (indels) (Fig. 1), alongside 132 SVs (Figs. 2A and 3A). Assessment of tumor mutational signatures identified a variety of constellations, which, however, lacked an apparent EBV-driven predominance (Supplementary Fig. 2). Polymorphic cases harbored significantly more subclones (ANOVA: *P* = 0.045), while showing no enrichment in the number of mutations (albeit at similar relative tumor cell content following microdissection/macrodissection) (Supplementary Fig. 3).

### Significantly mutated candidate driver genes in NFκB, WNT, and IL6/JAK/STAT dominate the mutational landscape of EBV + DLBCL (NOS)

**Fig. 4:**
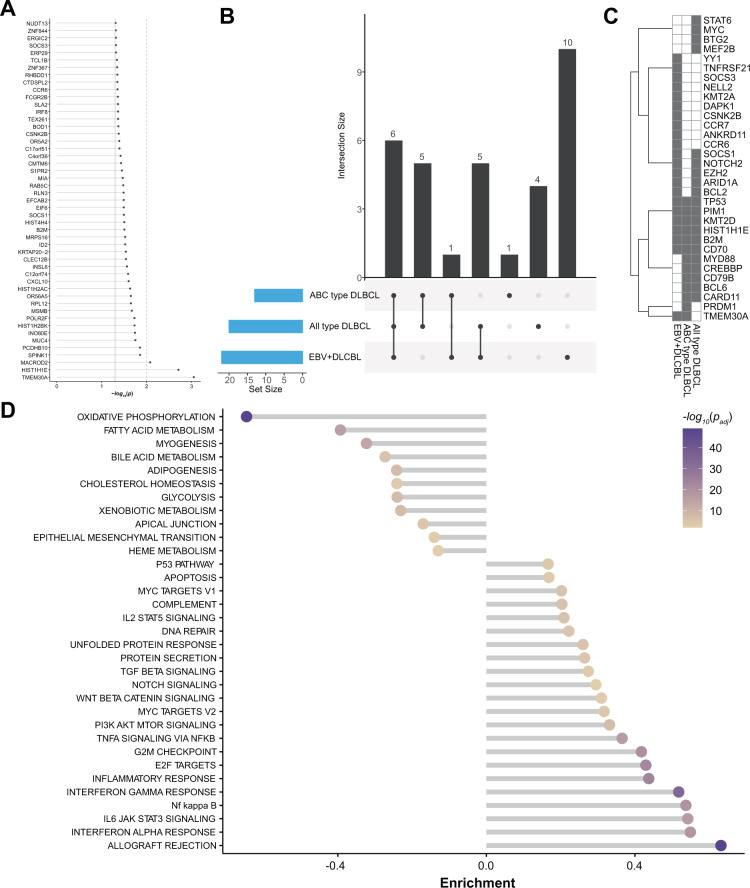
MutSigCV analysis for variant calling. **A** Significance levels for all EBV + DLBCL (NOS) MutSigCV genes (*P* < 0.05) and UpSet plot (**B**) showing the overlap of MutSigCV genes using EBV + DLBCL (NOS), all-type DLBCL and ABC-type DLBCL data; **C** shows the overlap between the three tumor entities. **D** HALLMARK gene sets enrichment for network diffusion analysis of significant MutSigCV genes (MutSigCV *P* < 0.05).

We next screened for significantly mutated genes and potential oncogenic drivers via MutSig2CV (Fig. 4A). A gene set enrichment analysis allocated recurrent mutations to their respective biological pathways (Supplementary Fig. 4). Hereby, we unveiled a potentially perturbing role of several recurrent alterations in NFκB (75%; *CSNK2A2*, *CARD10*, *LYN*, *SYK*), IL6/JAK/STAT (38%; *SOCS1*, *CXCL10*, *PIM1*, *SOCS3*, and *STAT3*) as well as WNT signaling (62%; *FRAT1*, *FRAT2*, *LRP5*). A network propagation approach (Fig. 4B) using potential driver mutations as seeds uncovered a significant enrichment in the mutational signature of EBV + DLBCL (NOS) for immunological processes, including allograft rejection, interferon-alpha and gamma response, as well as IL6/JAK/STAT3 signaling. Yet, cellular metabolism processes like oxidative phosphorylation as well as fatty acid metabolism were significantly spared from oncogenic mutations. In order to evaluate our cohort in the context of the molecularly restructured landscape of DLBCL, we cross-referenced our WGS mutational data with the recently described molecular clusters proposed by Chapuy et al. and thereby found no significant affiliation to a singular subgroup but rather a wide distribution onto the different clusters (Supplementary Fig. 5).

### Additional insights into EBV + DLBCL (NOS) mutational landscape revealed by deep targeted-amplicon-based sequencing

To expand on our data derived from WGS, we performed targeted resequencing on a tumor-only extension cohort comprising the patient samples from WGS for validation purposes. A custom in-house AmpliSeq panel for tNGS, encompassing genes previously implicated in B-cell lymphoma pathogenesis combined with mutational targets first identified from the WGS cohort, identified putative oncogenic driver mutations in 40/47 patients (Fig. 3B, F–I, lollipop plots) and detected 250 mutations (resulting in a median of four mutations/sample), including 245 SNVs and 5 insertions and deletions (indels). The mutational signature was dominated by variants affecting *ARID1A* (45%), *KMT2A* (32%), *ANKRD11* (32%), *NOTCH2* (32%), *KMT2D* (30%) *CCR6* (15%) in frequencies, differing significantly from previous findings in EBV-negative DLBCL. Upon comparative analysis of WGS and targeted resequencing data, we were able to demonstrate a concordance rate of 84% (21/25) of mutational calls (when considering genes with a mutational frequency >10% in our present cohort).

### *CCR6*, *CCR7*, *DAPK1*, *TNFRSF21*, and *YY1* mutations occur recurrently and appear to be specific for EBV + DLBCL (NOS)

To further elucidate the genomic taxonomy of B-cell malignancies and to clarify the mutational similarities and distinctions between EBV + DLBCL (NOS) and the closely related unselected ABC-type DLBCL) as well as (all-type) de novo DLBCL (NOS (*n* = 1151^45–48^), we compared candidate mutational drivers.

In accordance with previous reports, *ARID1A* (45%), *KMT2A* (32%), *ANKRD11* (32%), *NOTCH2* (32%), and *KMT2D* (30%) were recurrently mutated in our EBV + DLBCL (NOS) cohort, albeit at partially elevated frequencies compared with EBV-negative DLBCL. Intriguingly, our integrated WGS and tNGS data analysis found *CCR6* (15%), *DAPK1* (15%), *TNFRSF21* (13%), and *CCR7* (11%) mutations as reoccurring and specific features of EBV + DLBCL (NOS), when compared with other subtypes of DLBCL. Further genes that were significantly enriched for mutations compared to EBV- DLBCL (NOS) included *YY1*, *SOCS3*, *NELL2*, *CSNK2B*. Our data revealed similarities to the recently published cohort of strictly polymorphic EBV + DLBCL NOS (Poly-EBV-L) by Sarkozy et al. in terms of oncogenic mutations in *SOCS1*, *STAT6* and *KMT2D*, and differences with respect to the mutational frequencies in *GNA13, CSF2RB, CSMD3, CD58* and *PRKDC* (prominent in Poly-EBV-L), the typical DLBCL mutations in *ARID1A*, *KMT2A*, *ANKRD11*, and *NOTCH2 and lastly the* exclusive mutations for EBV + DLBCL (NOS) in the genes *CCR6*, *CCR7*, *DAPK1, TNFRSF21, CSNK2B,* and *YY1*^18^ (Fig. 4C)*.*

### 6q deletions identified by WGS and FISH are a recurrent feature of EBV + DLBCL (NOS)

Structural variants detected by WGS are summarized in Supplementary Table 3 and depicted in Supplementary Fig. 6. Upon additional interrogation for CNVs, we identified several recurrent alterations, most prominent of which was a 6q deletion of variable extent (Fig. 2A, B; 5/8 cases), that encompassed known driver genes of B-cell lymphomagenesis *PRDM1* and *A20* in 4/5 cases.

FISH analysis of all cases having available tissue sections in both the WGS and the extension cohort, identified 6q deletions in 14/32 (44%) cases (concurrence rate of 100% between FISH and WGS) (Fig. 2C, D). Interestingly, these cases harbored significantly fewer mutations for the oncogenic drivers *ANKRD11* and *NOTCH2* (Fig. 3E), yet no further differences with respect to clinical or pathological characteristics were found in patients with 6q deletion (Supplementary Table 4). Other recurrent and previously described cytogenetic alterations in EBV + DLBCL (NOS) include 17p del (2/8) resulting in a deletion of *TP53*.

### Survival analysis

Upon combined analysis of molecular and clinical data, we interrogated genomic alterations regarding their clinical impact. Due to the explorative nature of the study, Bonferroni’s correction for multiple testing was omitted. Hereby, we identified a significant impact (*P* < 0.05) of a distinct molecular subtype exhibiting combined alterations of *ARID1A* and *DAPK1*, which correlated with inferior overall survival. Moreover, mutations in *ANKRD11* were identified as indicators of superior relapse-free but not overall survival (Fig. 3C, D). These effects were still visible as trends bordering on statistical significance when restricting the analysis on R-CHOP-treated patients. By means of a Chi-squared-test, we determined the statistical independence of mutations in *ARID1A* (*P* = 0.3782), *DAPK1* (*P* = 0.2615), and *ANKRD11* (*P* = 0.369) from 6q deletion status.

## Discussion

Our whole-genome and targeted resequencing study in the hitherto largest cohort of EBV + DLBCL (NOS) in which all cases underwent rigorous central hematopathological panel review and cytogenetic workup prior to inclusion, we find a significant perturbing role of several recurrent alterations in NFκB, WNT, and IL6/JAK/STAT signaling. In line with previous publications, the overall mutational frequency was lower than in EBV-negative de novo DLBCL (NOS), which is compatible with the hypothesis that EBV-driven pathogenesis relies on a limited number of additional driver mutations and requires fewer oncogenic events than EBV-negative malignancies in order to shape a similar phenotype^49^. Further, our results support the preliminary notion on EBV + DLBCL (NOS) proposed by Liu et al., derived from a small Asian cohort, which emphasized a heterogeneous mutational profile with only few recurrent mutational events^15^. From our study, we deduce two essential insights into the molecular pathobiology of EBV + DLBCL (NOS).

First, we have delineated its mutational landscape, which is partially consistent with the literature as outlined by preliminary studies, recently published by Sarkozy and Zhou in terms of oncogenic mutations in *KMT2D*, *CD58, BCL6,* and *NOTCH2*^16,18^. Previously reported mutations in *TET2, DNMT3A*, and *TNFRSF14* were, however, not detectable in a significant subset of WGS patients. These observations do however require interpretation in the light of diverging compositions of selected NGS panels and the potential geographic heterogeneity between Asian and western DLBCL cohorts^17^. We identified no significant impact of mono- or polymorphic subtype of EBV + DLBCL on mutational signatures or mutational burden and no overall EBV-associated patterns or signatures were observed. Polymorphic lymphomas were, however, shown to harbor significantly more subclones, reflecting the morphological impression on a mutational level.

Our current investigation expands on previous efforts, not only through the largest number of cases studied so far, or a tumor-normal controlled WGS, rather than a tumor-only exome-focused approach but through the inclusion of both monomorphic as well as polymorphic (recurrently exhibiting Hodgkin and/or Reed/Sternberg cells) EBV + DLBCL (NOS). The genomic taxonomy of B-cell malignancies is, however, significantly refined in the current study through the comparative analysis of our data and the mutational studies in both unselected de novo DLBCL (NOS) as well as ABC-type DLBCL, as we reveal the mutational spectrum of aggressive EBV-driven lymphomas to exclusively encompass oncogenic mutations in *CCR6*, *CCR7*, *DAPK1*, *TNFRSF21, CSNK2B*, and *YY1*. Previous studies propose an oncogenetic role for mutations affecting *CCR6*. This is especially true for MALT-type lymphoma, where mutations are implicated in defective β-arrestin-mediated receptor desensitization and internalization, which is hypothesized to result in impaired regulation of transduction of extracellular stimulation into intracellular signaling^50^. Further studies implicated a (mutational) deregulation of the CCL20/CCR6 axis not only in inflammatory and infectious diseases but in disease progression across a variety of cancers, including pancreatic, colorectal, and breast cancer alongside hepatocellular carcinoma and others^51^. Moreover, *CCR7* induction in EBV-infected cells was recurrently proposed to enable homing of lymphoid cells to secondary lymphoid tissue, where the virus in turn propagates infection or establishes latency, thereby driving lymphomagenesis^52^. It is further tempting to speculate on a potential role of oncogenic *CCR7* mutations in EBV + DLBCL (NOS), prompting cellular proliferation and migration upon the binding of the cognate chemokine receptors, as was recently shown in breast cancer and other solid tumors^53^. Of note, we describe oncogenic mutations in *DAPK1* as an exclusive feature of EBV + DLBCL (NOS). In a recent study, hypermethylation of *DAPK1*, speculatively resulting in a similar loss of function of DAPK1 as a regulatory partner of *TP53* was shown to predict inferior clinical outcome^54^. Intriguingly, impairment of *TNFRSF21*/*DR6* was previously implicated in increased cellular division and reduced apoptosis rates in B-cells and T-cell malignancies, including angioimmunoblastic T-cell lymphoma, another, recurrently EBV-driven type of cancer^55,56^. The known oncogenic driver *YY1* was previously implicated in DLBCL pathogenesis irrespective of EBV status. Its overexpression was found to result in B-cell transformation and tumor progression, while mutations affecting *CSNK2B*, as another exclusive feature of EBV + DLBCL (NOS), remain insufficiently characterized to date^57^. Further, we sought to allocate our EBV + DLBCL (NOS) WGS cohort within the system of molecular clusters proposed by Chapuy et al. by means of cross-referencing evaluation of mutational patterns. This, however, yielded no predominant association with a singular cluster, most likely marking EBV positivity as an independent oncogenic driver, not restricted to a particular DLBCL subtype. We hereby offer substantial and novel insights into the molecular pathogenesis of this rare entity, potentially providing novel therapeutic targets, which now require functional validation.

Second, analyzing the entity on the scale of whole genomic structural variants and CNVs and thereby expanding on previous array comparative genomic hybridization findings by our group, we were able to identify recurrent 6q deletions. These observations are in line with the post-germinal phenotype of EBV + DLBCL (NOS) given that recurrent 6q deletions, affecting both *PRDM1* and *A20*, have previously been implicated in ABC-type DLBCL pathogenesis as adverse prognosticators^58^. Intriguingly, we found 6q del cases to harbor significantly fewer mutations in oncogenic drivers such as *ANKRD11* and *NOTCH2*, hinting at an independent oncogenic effect of this aberration, requiring even fewer additional genomic hits than 6q wild-type EBV + DLBCL (NOS) to achieve the malignant phenotype of the disease. This has been described before in EBV-negative ABC-type DLBCL, where the loss of PRDM1/BLIMP-1 was revealed to result in significantly reduced expression of genes implicated in plasma-cell differentiation while favoring the expression of genes involved in B-cell receptor signaling and proliferation^58^.

Integrative analysis of mutational and clinical data revealed combined alterations of *ARID1A* and *DAPK1* as prognosticators of adverse overall survival, while mutations in *ANKRD11* appeared to predict superior relapse-free, but not overall survival. These observations were apparently independent of 6q deletion status, a previously described adverse prognosticator in all-type DLBCL undergoing immunochemotherapy^59^ These preliminary observations do, however, require further validation in a prospective cohort of patients, preferably gathered within the context of a clinical trial.

Limitations of our current study include its retrospective design, implying the potential for fragmentary clinical data. Moreover, paired germline DNA samples for a larger group of EBV + DLBCL (NOS) would have been preferable in order to strengthen the exploratory subgroup of the cohort in our WGS approach. Identification of novel mutational hotspots may have been hindered by the limited number of samples in this group and further studies are needed to validate our findings. Further, the panel, employed in the analysis of our tumor-only extension cohort was limited, resulting in the identification of potentially oncogenic mutations in a mere 40/47 cases of EBV + DLBCL (NOS). Future studies should preferentially rely on a larger panel of genes or whole-exome sequencing.

In summary, we delineate the mutational landscape of EBV + DLBCL (NOS) and comprehensively explore its similarities and distinctions, compared with other types of DLBCL, including several exclusive oncogenic drivers. These provide a valuable starting point for targeted therapy approaches in this entity. In addition, we describe large deletions on chromosome 6 to be a highly recurrent cytogenetic feature of this rare entity, decisively shaping its near-terminally differentiated B-cell phenotype.

## Supplementary information

Supplementary Figure 1

Supplementary Figure 2

Supplementary Figure 3

Supplementary Figure 4

Supplementary Figure 5

Supplementary Figure 6

Supplementary Figure 7

Supplementary Figure 8

Supplemental Material

21-BCJ-155R Checklist
