# Supplementary figures and images for: Genomic insights into the pathogenesis of Epstein–Barr virus-associated diffuse large B-cell lymphoma by whole-genome and targeted amplicon sequencing
Source: Blood Cancer J. 2021 May 26;11(5):102. doi: 10.1038/s41408-021-00493-5 (PMC8155002; doi:10.1038/s41408-021-00493-5)

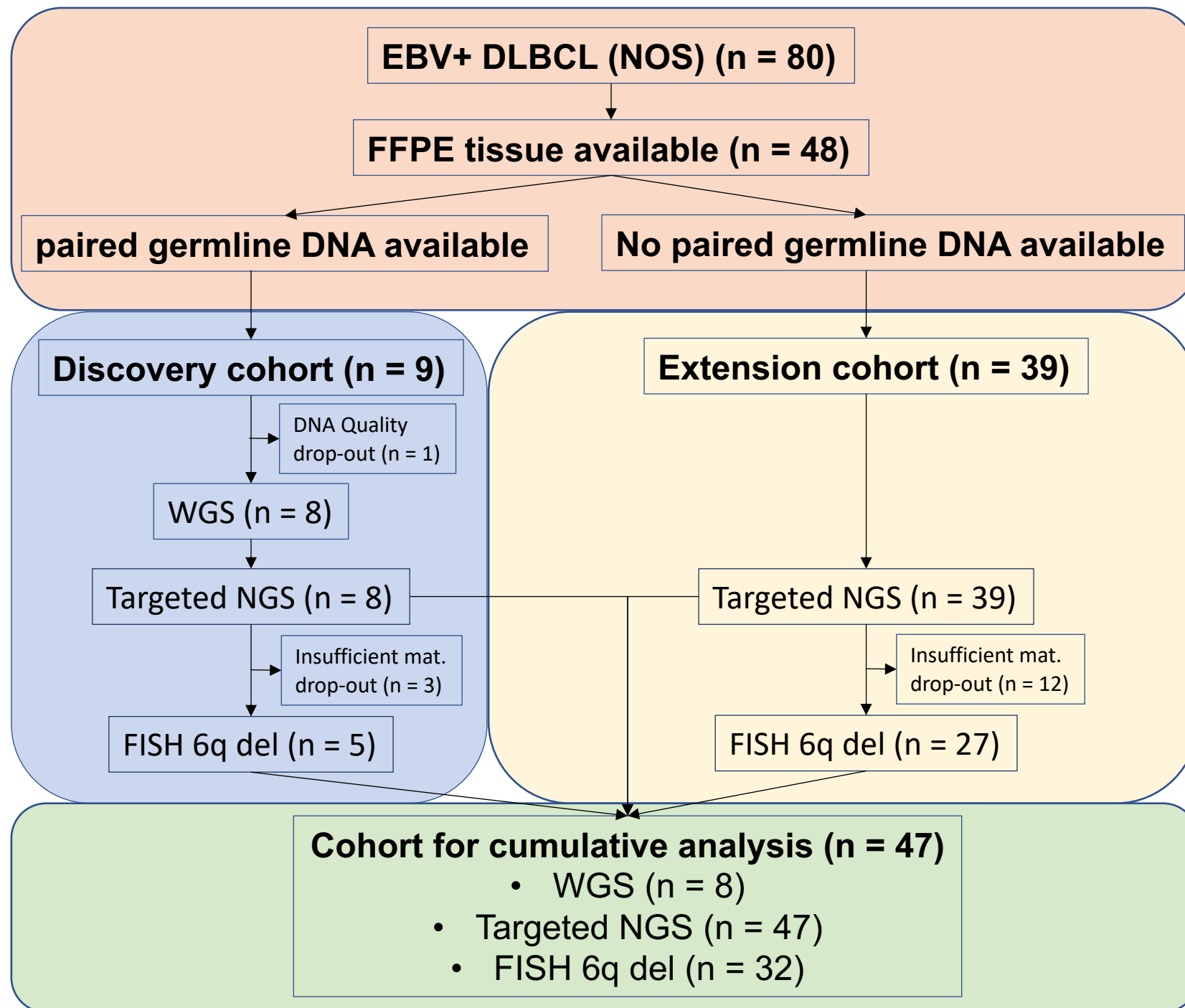

Supplement: Supplementary file 1 — Supplementary Figure 1 [file 41408_2021_493_MOESM1_ESM.pdf]

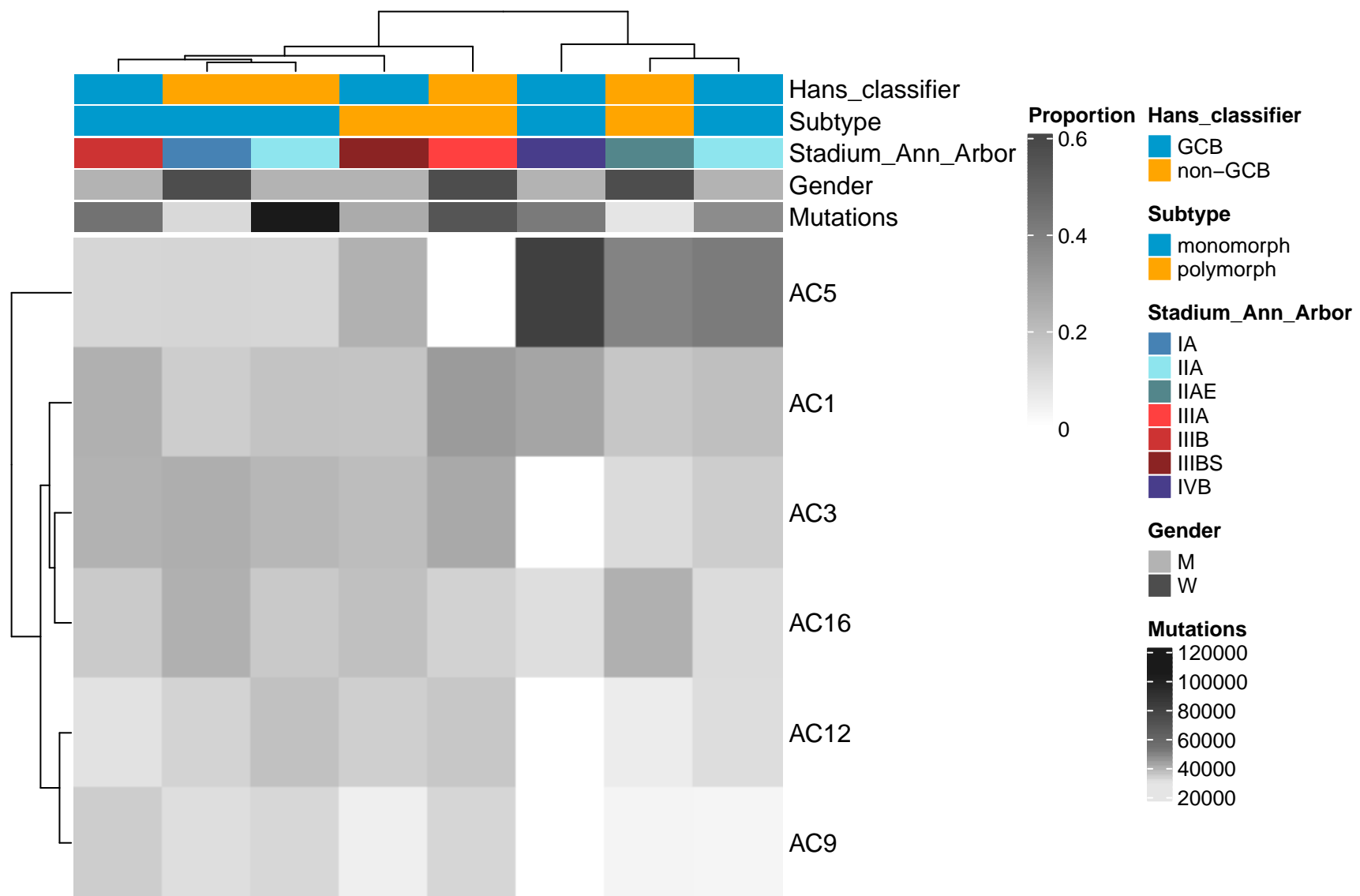

Supplement: Supplementary file 2 — Supplementary Figure 2 [file 41408_2021_493_MOESM2_ESM.pdf]

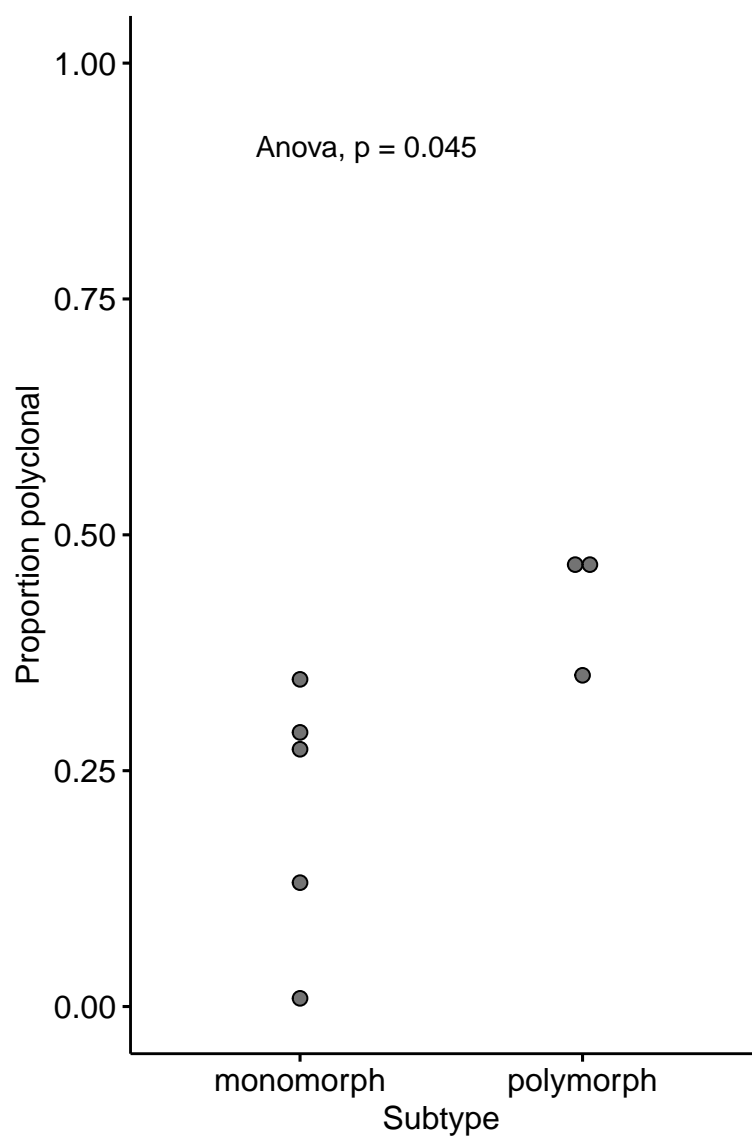

Supplement: Supplementary file 3 — Supplementary Figure 3 [file 41408_2021_493_MOESM3_ESM.pdf]

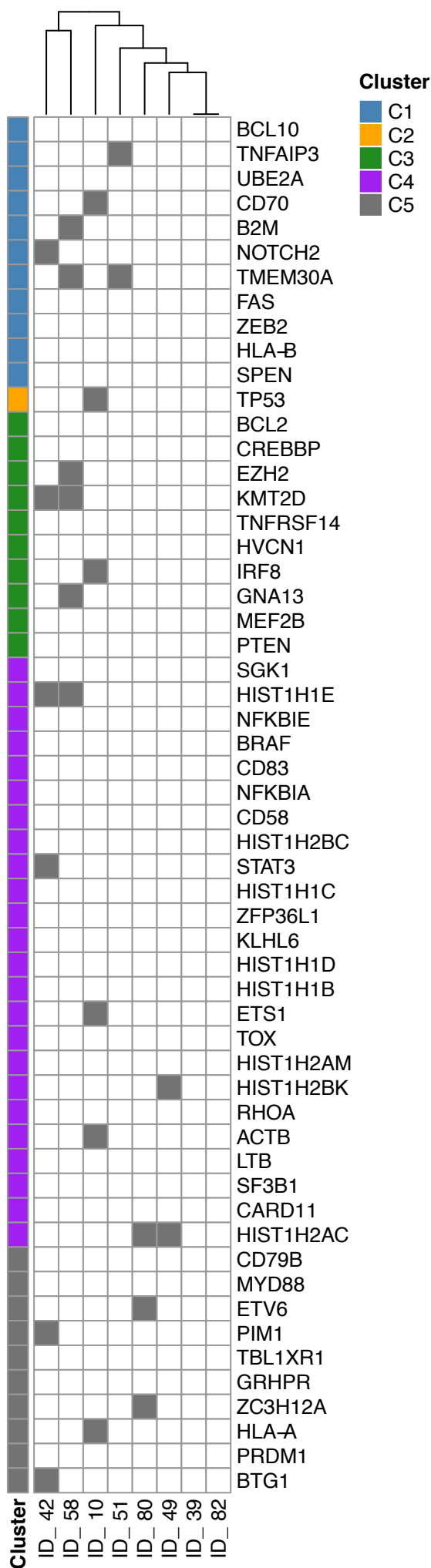

Supplement: Supplementary file 5 — Supplementary Figure 5 [file 41408_2021_493_MOESM5_ESM.pdf]

**A**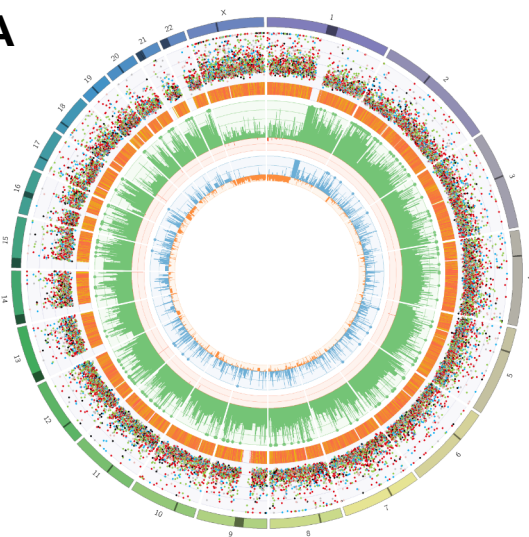**B**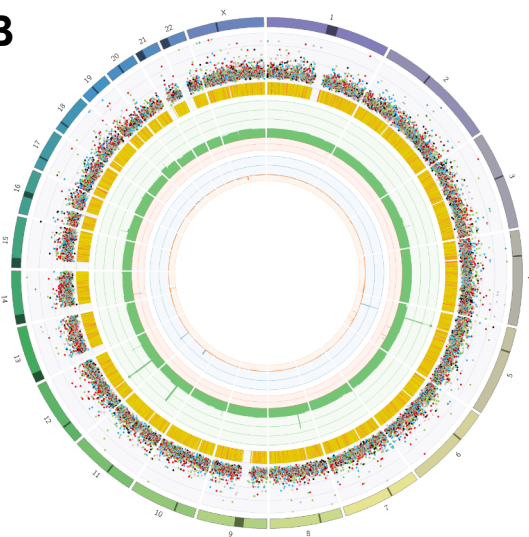**C**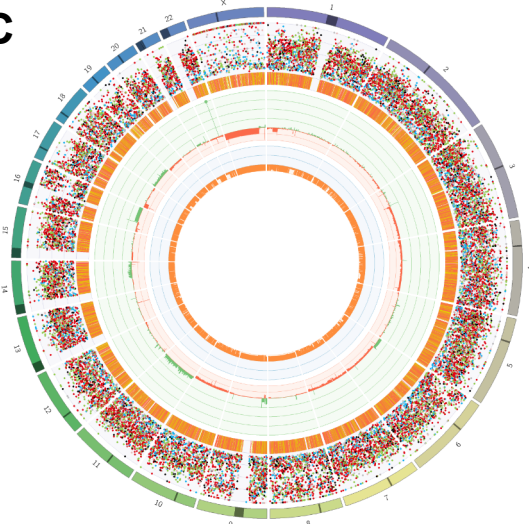**D**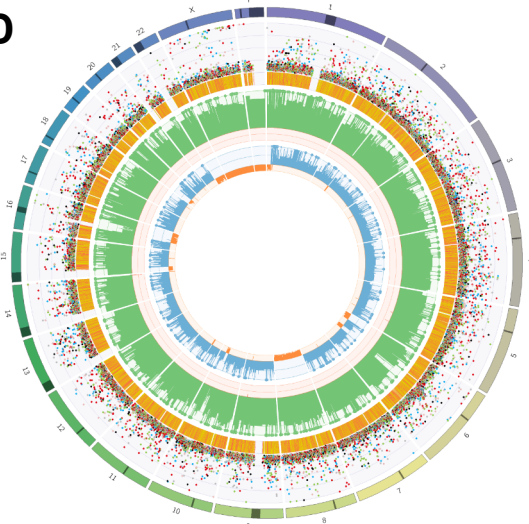**E**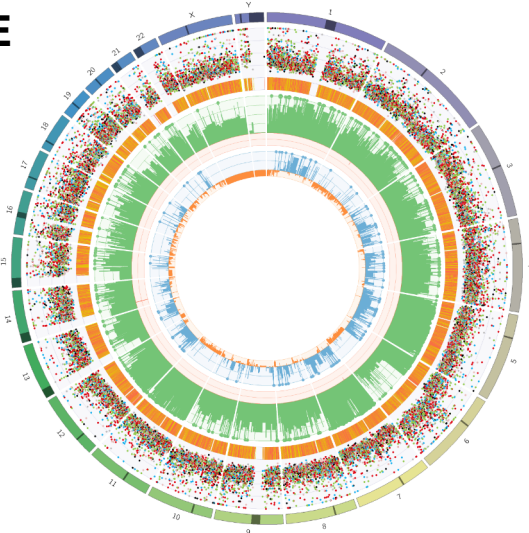**F**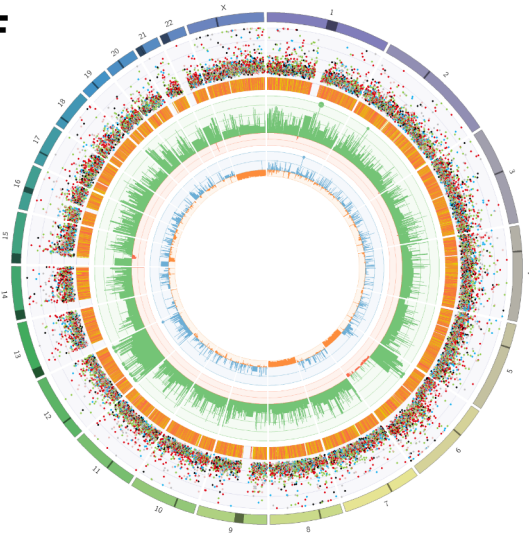**G**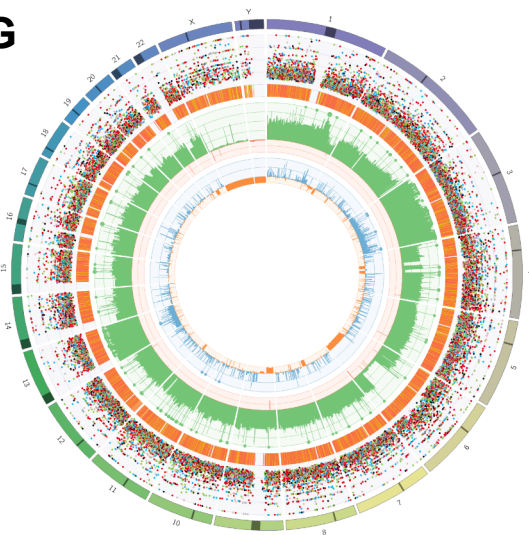**H**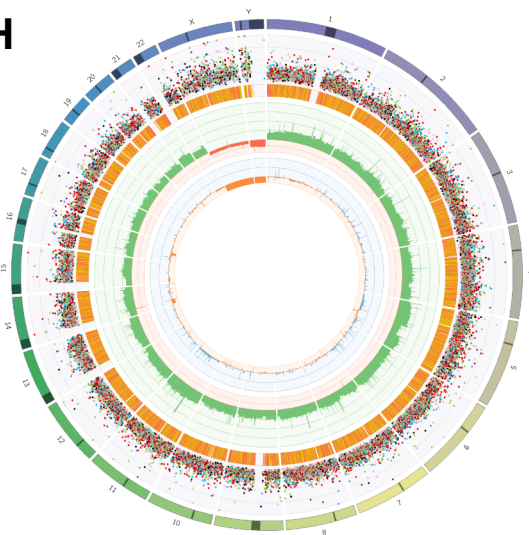

Supplement: Supplementary file 6 — Supplementary Figure 6 [file 41408_2021_493_MOESM6_ESM.pdf]

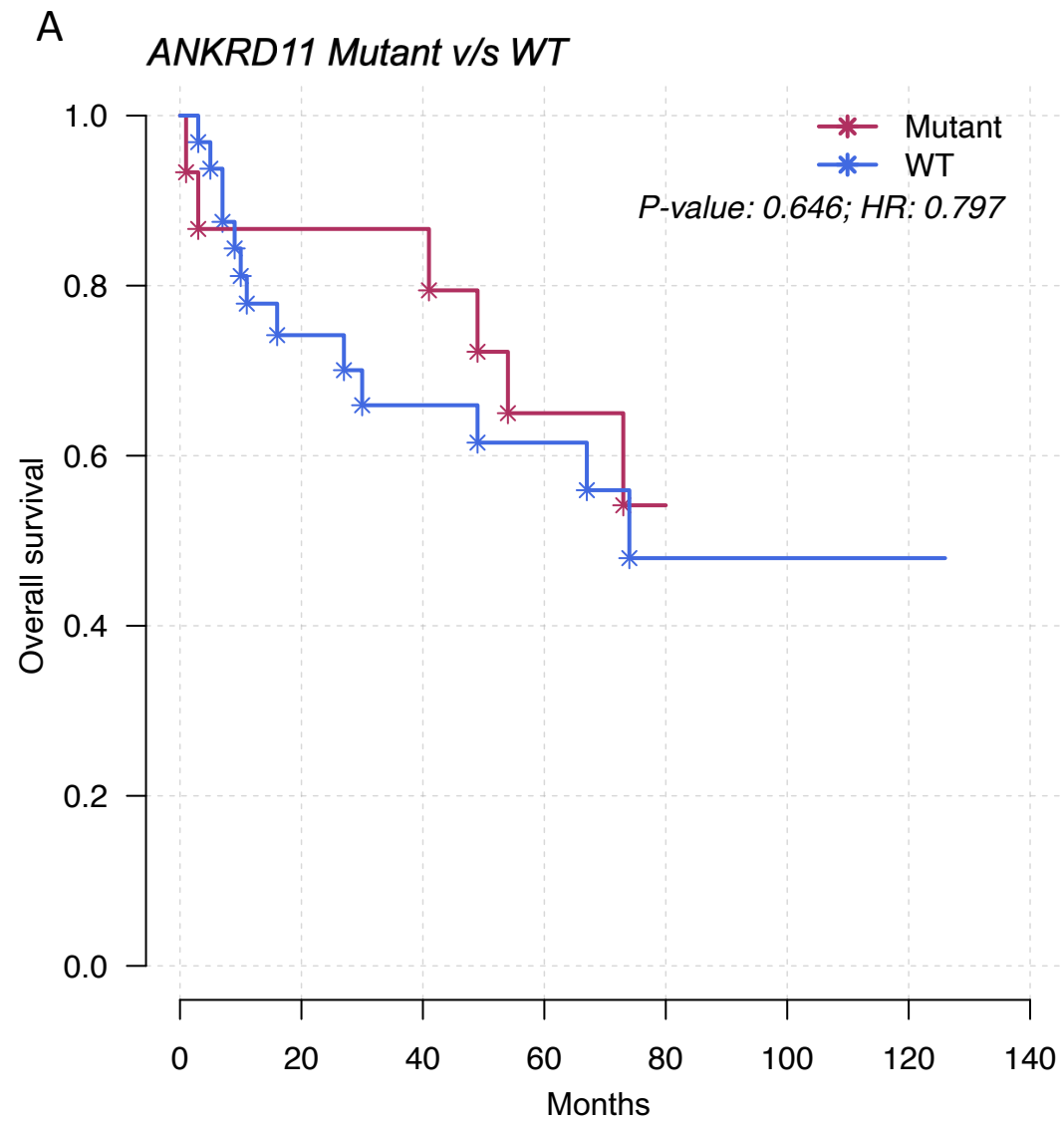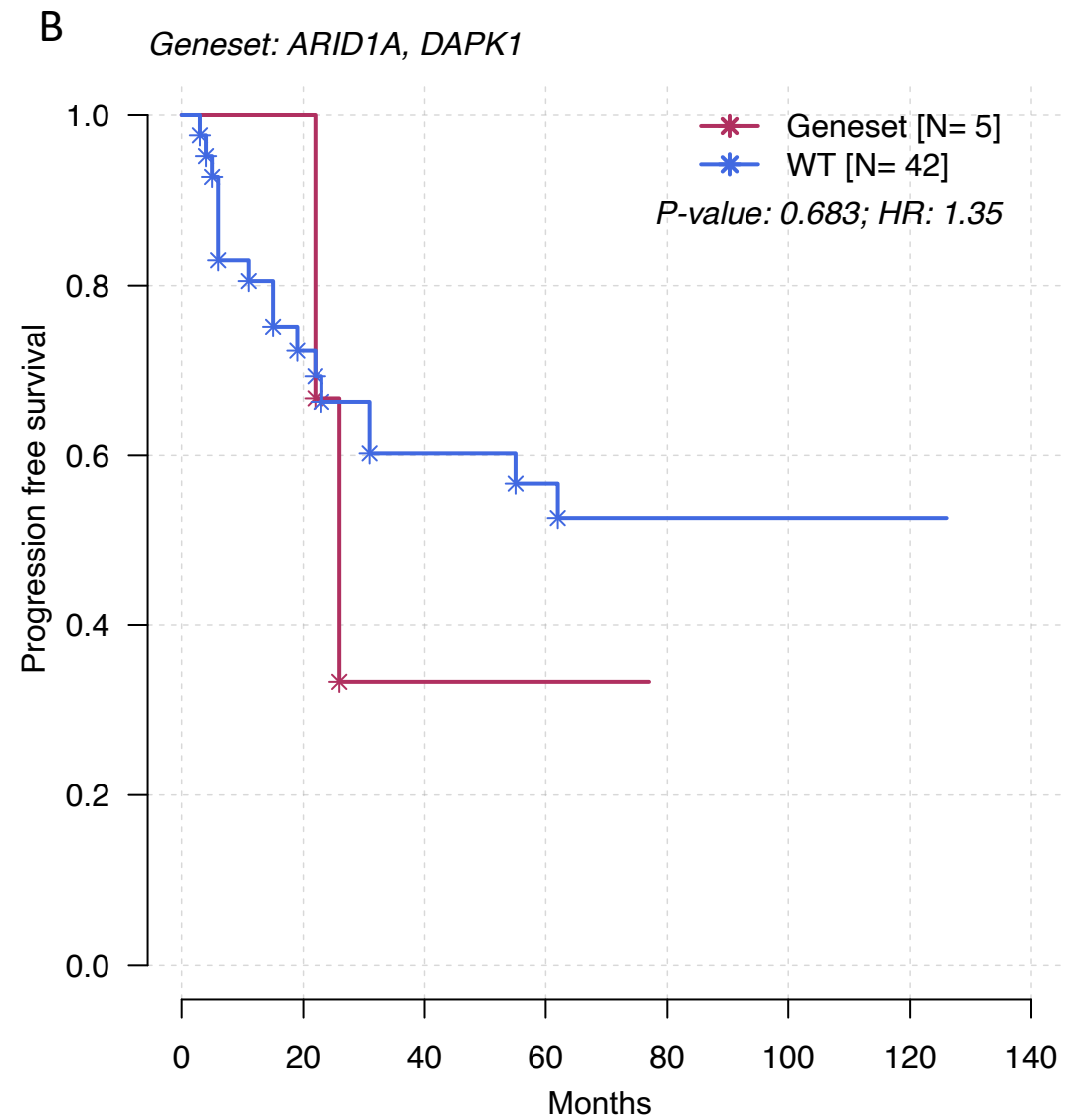

Supplement: Supplementary file 7 — Supplementary Figure 7 [file 41408_2021_493_MOESM7_ESM.pdf]

A

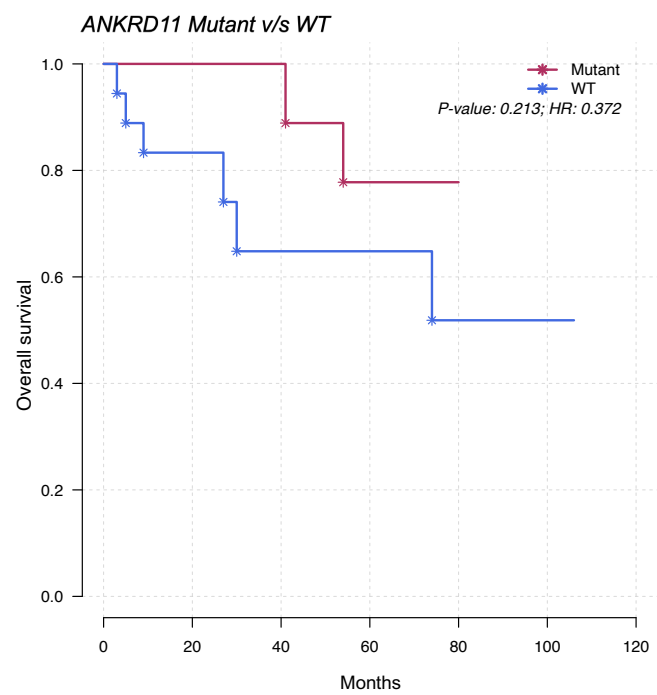

B

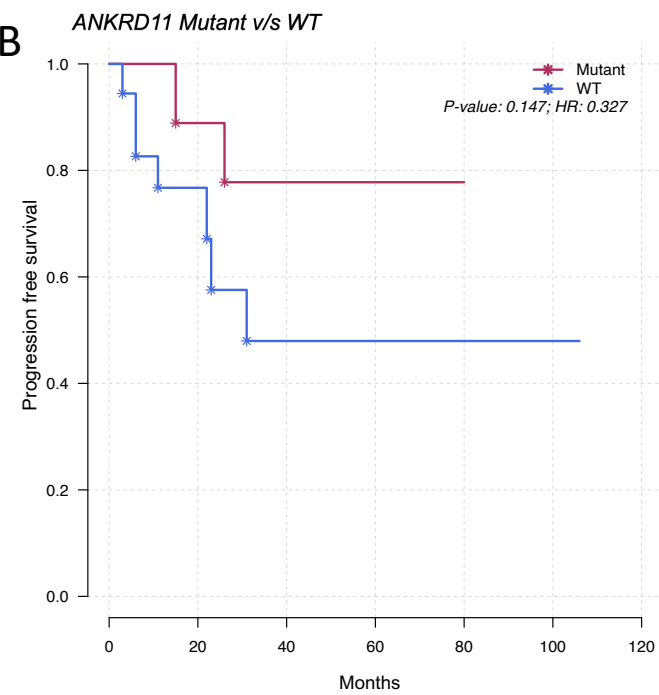

C

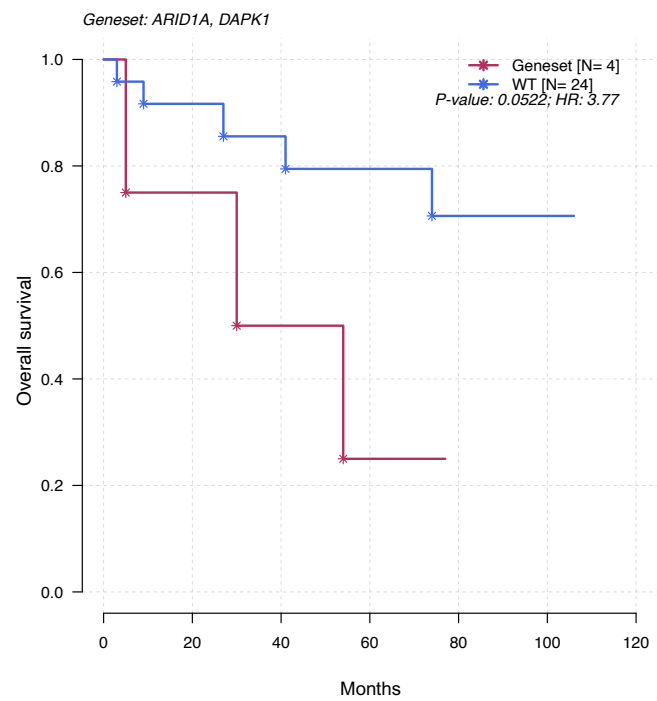

D

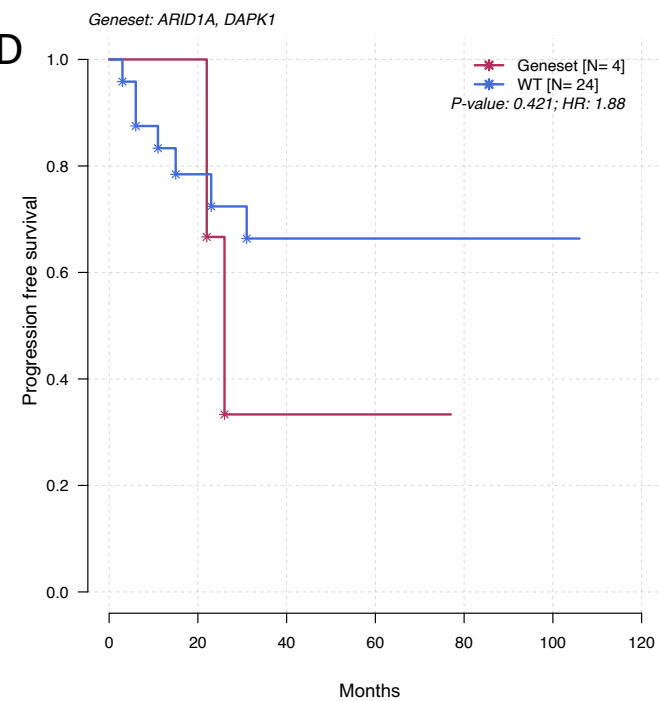

Supplement: Supplementary file 8 — Supplementary Figure 8 [file 41408_2021_493_MOESM8_ESM.pdf]
