# Supplementary material for: Genomic insights into the pathogenesis of Epstein–Barr virus-associated diffuse large B-cell lymphoma by whole-genome and targeted amplicon sequencing
Source: Blood Cancer J. 2021 May 26;11(5):102. doi: 10.1038/s41408-021-00493-5 (PMC8155002; doi:10.1038/s41408-021-00493-5)
Supplement: Supplementary file 4 — Supplementary Figure 4 [file 41408_2021_493_MOESM4_ESM.pdf]

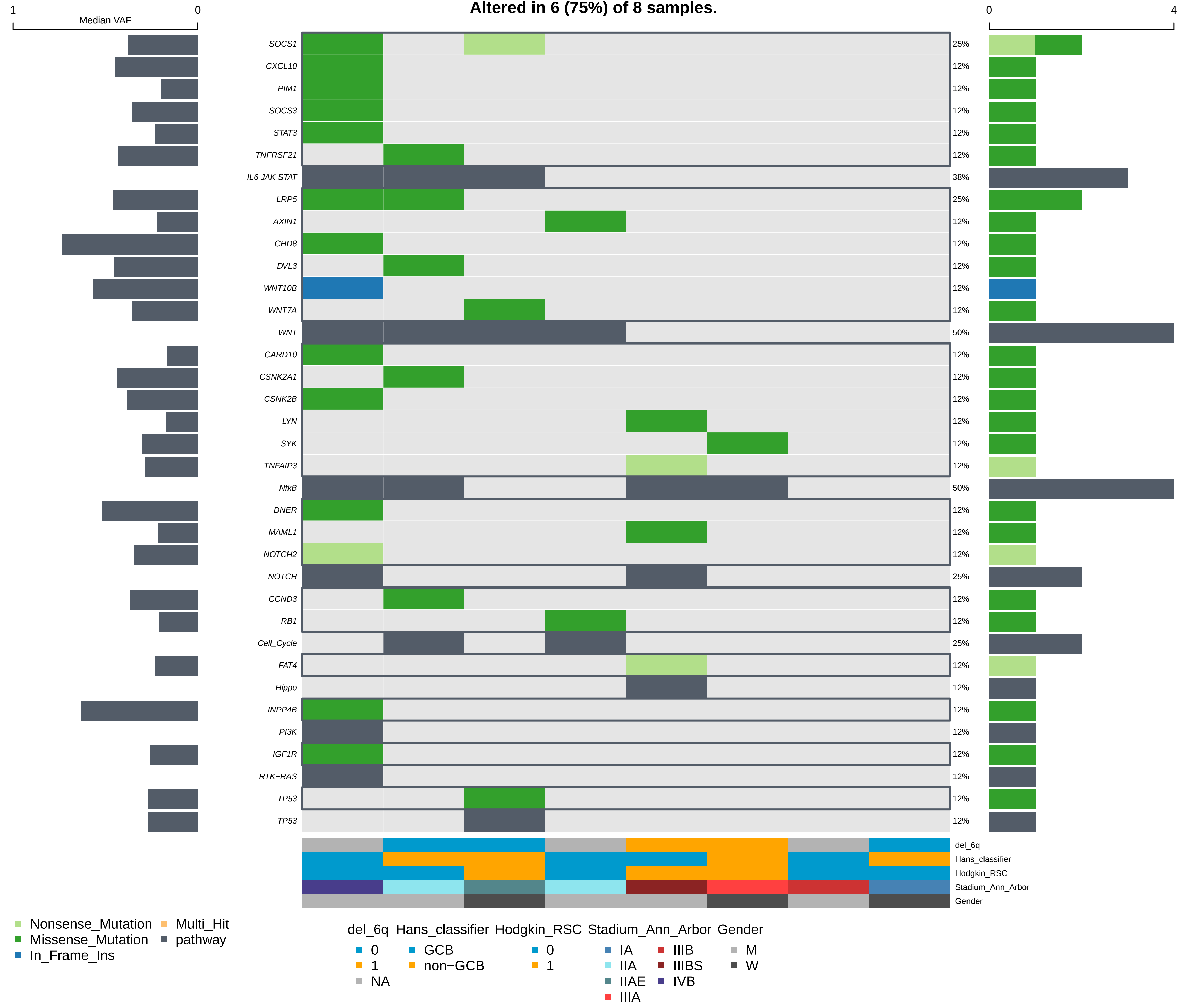
Altered in 6 (75%) of 8 samples.
Median VAF
1
0
0
4
SOCS1 25%
CXCL10 12%
PIM1 12%
SOCS3 12%
STAT3 12%
TNFRSF21 12%
IL6 JAK STAT 38%
LRP5 25%
AXIN1 12%
CHD8 12%
DVL3 12%
WNT10B 12%
WNT7A 12%
WNT 50%
CARD10 12%
CSNK2A1 12%
CSNK2B 12%
LYN 12%
SYK 12%
TNFAIP3 12%
NfkB 50%
DNER 12%
MAML1 12%
NOTCH2 12%
NOTCH 25%
CCND3 12%
RB1 12%
Cell_Cycle 25%
FAT4 12%
Hippo 12%
INPP4B 12%
PI3K 12%
IGF1R 12%
RTK−RAS 12%
TP53 12%
TP53 12%
del_6q
Hans_classifier
Hodgkin_RSC
Stadium_Ann_Arbor
Gender
Nonsense_Mutation
Missense_Mutation
In_Frame_Ins
Multi_Hit
pathway
del_6q
0
1
NA
Hans_classifier
GCB
non−GCB
Hodgkin_RSC
0
1
Stadium_Ann_Arbor
IA
IIA
IIAE
IIIA
IIIB
IIIBS
IVB
Gender
M
W
