# Supplementary material for: Genomic insights into the pathogenesis of Epstein–Barr virus-associated diffuse large B-cell lymphoma by whole-genome and targeted amplicon sequencing
Source: Blood Cancer J. 2021 May 26;11(5):102. doi: 10.1038/s41408-021-00493-5 (PMC8155002; doi:10.1038/s41408-021-00493-5)
Supplement: Supplementary file 9 — Supplemental Material [file 41408_2021_493_MOESM9_ESM.docx]

**Supplement:**

**Supplementary materials and methods:**

Diagnosis was confirmed by two experienced hematopathologists (ACF & HM) in accordance with the current edition of the WHO classification of tumors of the hematopoietic and lymphoid tissues ^1^.

Antibodies and positivity cut-offs employed in the current study are summarized in **Supplementary Table 1**. Fluorescence *in situ* hybridization (FISH) for *MYC* and chromogenic *in situ* hybridization for EBER were performed, as described ^2, 3^.

Clinical characteristics were collected from the original files, including revised international prognostic index (R-IPI)^4^ and survival data were anonymously recorded alongside hematopathological assessments and genomic analyses. Extent of disease was routinely evaluated according to the Cotswold modifications of the Ann Arbor classification ^5^. Most patients included in this study were previously investigated as part of a previous clinicopathological study ^6^. Patients had given written informed consent regarding routine diagnostic and academic assessment of their biopsy specimen at the Reference center for Hematopathology and transfer of their clinical data. Unfortunately, there was no comparable material left from a previous array CGH and Sanger sequencing study on EBV+ DLBCL (NOS) conducted at our institution ^7^.

**Supplementary Tables:**

**Supplementary Table 1.** Antibodies and positivity cut-offs employed in the current study

| **Antibody** | **Supplier** | **Clone** | **Positivity cut-off** |
| --- | --- | --- | --- |
| Bcl2  | Lab Vision | 100/D5 | 30% |
| Bcl6 | Dako | BG-B6p | 30% |
| CD10 | Menarini | 56C6 | 30% |
| CD20 | Dako | L26 | - |
| CD30 | Dako | BerH2 | 10% |
| MUM-1 (Irf4) | Dako | Mum 1P | 30% |
| Ki-67 | Dako | Mib-1 | - |

**Supplementary Table 2.** In-house custom AmpliSeq panel (Thermo Fisher Scientific, Waltham, Massachusetts, USA) for targeted amplicon sequencing, encompassing all coding exons of 43 genes

| **Gene** | |
| --- | --- |
| *ANKRD11* | *HIST1H1E* |
| *ARID1A* | *ID3* |
| *ARID2* | *IRF4* |
| *B2M* | *KMT2A* |
| *BCL2* | *KMT2D* |
| *BCL6* | *MEF2B* |
| *BTG2* | *MYC* |
| *CARD11* | *MYD88* |
| *CCR6* | *NELL2* |
| *CCR7* | *NOTCH2* |
| *CD274* | *PIM1* |
| *CD58* | *PRDM1* |
| *CD70* | *SOCS1* |
| *CD79A* | *SOCS3* |
| *CD79B* | *STAT6* |
| *CREBBP* | *TCF3* |
| *CSNK2B* | *TLR9* |
| *CXCL10* | *TMEM30A* |
| *DAPK1* | *TNFRSF21* |
| *EZH2* | *TP53* |
| *FAS* | *YY1* |
| *FOXO1* |  |

**Supplementary Table 3.** Summary of structural variants detected by WGS

| **Study_ID** | **ploidy** | **polyclonalProp** | **msIndelsPerMb** | **msStatus** | **NumberSVs** |
| --- | --- | --- | --- | --- | --- |
| ID_10 | 4,1 | 0,4756 | 3,230849948 | MSS | 166 |
| ID_39 | 2,98 | 0,0087 | 6,884575026 | MSI | 26 |
| ID_42 | 1,92 | 0,2906 | 3,092339979 | MSS | 52 |
| ID_49 | 5,2 | 0,3468 | 2,390696048 | MSS | 81 |
| ID_51 | 5,1 | 0,4612 | 1,719482336 | MSS | 34 |
| ID_58 | 3,25 | 0,3511 | 1,376705142 | MSS | 0 |
| ID_80 | 4,15 | 0,2727 | 1,193074502 | MSS | 421 |
| ID_82 | 3 | 0,1313 | 2,058412032 | MSS | 233 |

**Supplementary Table 4.** Baseline clinicopathological characteristics in EBV+ DLBCL patients with/without cytogenetic 6q aberration.

| **Supplementary Table 1.** Baseline clinicopathological characteristics in EBV+ DLBCL patients with versus without cytogenetic 6q aberration. | | |
| --- | --- | --- |
| **Characteristics** | **6q deletion (n = 14)** | **6q wildtype (n =18)** |
| **Age (yrs.; median + range)** | 76.5 (51 – 81) | 68.5 (40 – 90) |
| **Sex** | | |
| Female | 5 (35.7%) | 8 (44.4%) |
| Male | 9 (64.3%) | 10 (55.6%) |
| **R-IPI** | | |
| 0 | 1 (7.1%) | 1 (5.6%) |
| 1-2 | 7 (50.0%) | 8 (44.4%) |
| >2 | 6 (42.9%) | 9 (50.0%) |
| **Stage (Ann Arbor)** | | |
| I | 2 (14.3%) | 4 (22.2%) |
| II | 6 (42.9%) | 5 (27.8%) |
| III | 5 (35.7%) | 3 (16.7%) |
| IV | 1 (7.1%) | 6 (33.3%) |
| **B-Symptoms** | | |
| Yes | 5 (35.7%) | 11 (61.1%) |
| No | 9 (64.3%) | 7 (38.9%) |
| **CD30 by immunohistochemistry** | | |
| Positive | 10 (71.4%) | 13 (72.2%) |
| Negative | 4 (28.6%) | 5 (27.8%) |
| **Extranodal sites** | | |
| 0 | 8 (57.1%) | 9 (50.0%) |
| 1-2 | 6 (42.9%) | 9 (50.0%) |
| **ECOG PS** | | |
| 0-1 | 5 (35.7%) | 8 (44.4%) |
| ≥2 | 9 (64.3%) | 10 (55.6%) |
| **LDH** | | |
| Normal | 6 (42.9%) | 8 (44.4%) |
| Elevated | 8 (57.1%) | 10 (55.6%) |
| **CNS involvement at diagnosis** | | |
| Yes | 1 (7.1%) | 2 (11.1%) |
| No | 13 (92.9%) | 17 (94.4%) |
| **Frontline therapy regimen** | | |
| CHOP-like | 9 (64.3%) | 14 (77.8%) |
| R-based | 12 (85.7%) | 17 (94.4%) |
| Others | 1 (7.1%) | 1 (5.6%) |
| Refusal of treatment | - | - |
| **Frontline therapy response rates** | | |
| CR | 9 (64.3%) | 13 (72.2%) |
| PR | 4 (28.6%) | 2 (11.1%) |
| SD | 1 (7.1%) | 2 (11.1%) |
| PD | - | 1 (5.6%) |
| EBV, Epstein Barr Virus; Yrs., years; CNS, central nervous system; LDH, Lactate dehydrogenase; ECOG; Eastern cooperative oncology group; PS, performance status; CHOP, cyclophosphamide, doxorubicin, vincristine, prednisolone; R, rituximab; Others, other regimen (e.g. Bendamustine) or palliative cytoreductive treatment. | | |

**Supplementary Figures:**

**Supplementary Figure 1.** A summary in the format of a flow (CONSORT) diagram depicting the selection of cases for the respective analyses alongside a description of drop-outs

**Supplementary Figure 2.** Evaluating WGS data for mutational signatures we identified a variety of constellations without an apparent EBV-driven predominance of a specific signature

**Supplementary Figure 3.** Polymorphic cases were however shown to harbor significantly more subclones (ANOVA: p = 0.045) while showing no enrichment for absolute mutations (albeit at similar relative tumor cell content following micro-/macro-dissection)

**Supplementary Figure 4.** Mutations grouped by signaling pathways and patients according to gene set enrichment analysis

**Supplementary Figure 5**. Cross-referencing WGS mutational data with the recently described molecular clusters proposed by Chapuy *et al.* no significant affiliation to a singular subgroup but rather a wide distribution onto the different clusters was encountered ^8^.

**Supplementary Figure 6.** Circos plots for each patient (A to H: ID_10, ID_39, ID_42, ID_49, ID_51, ID_58, ID_80, and ID_82) show the following tracks (from outermost to innermost circle): Chromosomes with gaps in the reference as dark shaded areas (e.g., centromeres, heterochromatin, missing short arms). The next track shows somatic variants with division into single nucleotide variants (SNVs; outer ring) and short insertions/deletions (inner ring, yellow and red for insertions and deletions, respectively). SNV frequencies have been corrected for tumor purity and scale from 0 to 1 (lines represent variant allele frequencies of 1, 0.75, 0.5, 0.25, and 0.0; coloring is according to the type of base change as described ^9^. The third track shows copy number changes adjusted for tumor purity (red: copy number less than 2; green: copy number greater than 2) and scales between 0 (complete loss) and 6 (high level gain). Copy numbers above 6 are shown is green dots. The innermost circle depicts the observed 'minor allele copy numbers’ across the chromosome ranging from 0 to 3 (expected minor allele copy number is 1) with amplifications of A and B alleles shown in blue and loss-of-heterozygosity events are shown in orange.

**Supplementary Figure 7.** Overall survival for ANKRD11 mutation status (A) and progression-free survival for the combined mutation set of ARID1A and DAPK1 (B) across the entire study cohort

**Supplementary Figure 8.** Impact of ANKRD11(A; B) and ARID1A/DAPK1 (C; D) mutation status on overall survival (A; C) and progression-free (B; D) in R-CHOP treated patients

**Supplementary References:**

1. Swerdlow SH, Campo E, Pileri SA, Harris NL, Stein H, Siebert R*, et al.* The 2016 revision of the World Health Organization classification of lymphoid neoplasms. *Blood* 2016 May 19; **127**(20)**:** 2375-2390.

2. Gebauer N, Bernard V, Gebauer W, Thorns C, Feller AC, Merz H. TP53 mutations are frequent events in double-hit B-cell lymphomas with MYC and BCL2 but not MYC and BCL6 translocations. *Leuk Lymphoma* 2015 Jan; **56**(1)**:** 179-185.

3. Montes-Moreno S, Odqvist L, Diaz-Perez JA, Lopez AB, de Villambrosia SG, Mazorra F*, et al.* EBV-positive diffuse large B-cell lymphoma of the elderly is an aggressive post-germinal center B-cell neoplasm characterized by prominent nuclear factor-kB activation. *Mod Pathol* 2012 Jul; **25**(7)**:** 968-982.

4. Sehn LH, Berry B, Chhanabhai M, Fitzgerald C, Gill K, Hoskins P*, et al.* The revised International Prognostic Index (R-IPI) is a better predictor of outcome than the standard IPI for patients with diffuse large B-cell lymphoma treated with R-CHOP. *Blood* 2007 Mar 01; **109**(5)**:** 1857-1861.

5. Lister TA, Crowther D, Sutcliffe SB, Glatstein E, Canellos GP, Young RC*, et al.* Report of a committee convened to discuss the evaluation and staging of patients with Hodgkin's disease: Cotswolds meeting. *J Clin Oncol* 1989 Nov; **7**(11)**:** 1630-1636.

6. Witte HM, Merz H, Biersack H, Bernard V, Riecke A, Gebauer J*, et al.* Impact of treatment variability and clinicopathological characteristics on survival in patients with Epstein-Barr-Virus positive diffuse large B cell lymphoma. *Br J Haematol* 2020 Jan 20.

7. Gebauer N, Gebauer J, Hardel TT, Bernard V, Biersack H, Lehnert H*, et al.* Prevalence of targetable oncogenic mutations and genomic alterations in Epstein-Barr virus-associated diffuse large B-cell lymphoma of the elderly. *Leuk Lymphoma* 2015 Apr; **56**(4)**:** 1100-1106.

8. Chapuy B, Stewart C, Dunford AJ, Kim J, Kamburov A, Redd RA*, et al.* Molecular subtypes of diffuse large B cell lymphoma are associated with distinct pathogenic mechanisms and outcomes. *Nat Med* 2018 May; **24**(5)**:** 679-690.

9. Alexandrov LB, Nik-Zainal S, Wedge DC, Aparicio SA, Behjati S, Biankin AV*, et al.* Signatures of mutational processes in human cancer. *Nature* 2013 Aug 22; **500**(7463)**:** 415-421.
